# Supplementary figures and images for: Deciphering the biosynthetic landscape of biofilms in glacier-fed streams
Source: mSystems. 2024 Dec 31;10(2):e01137-24. doi: 10.1128/msystems.01137-24 (PMC11834409; doi:10.1128/msystems.01137-24)

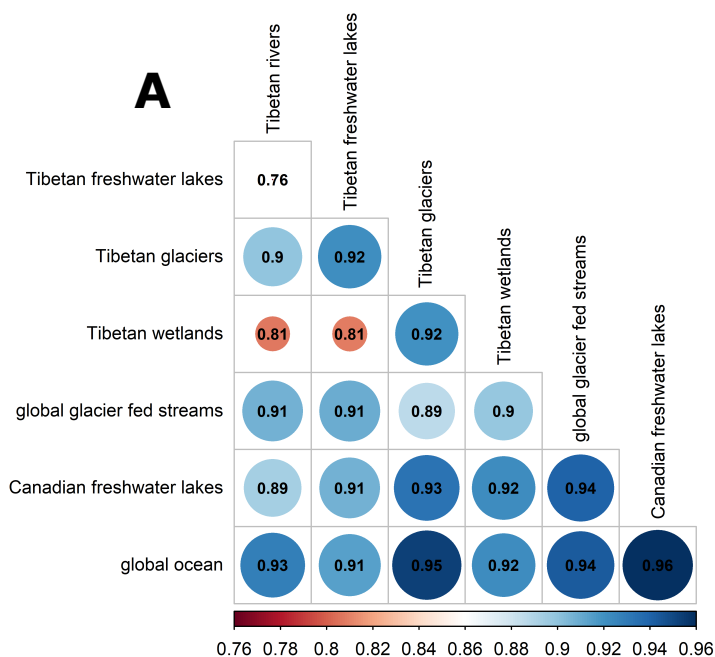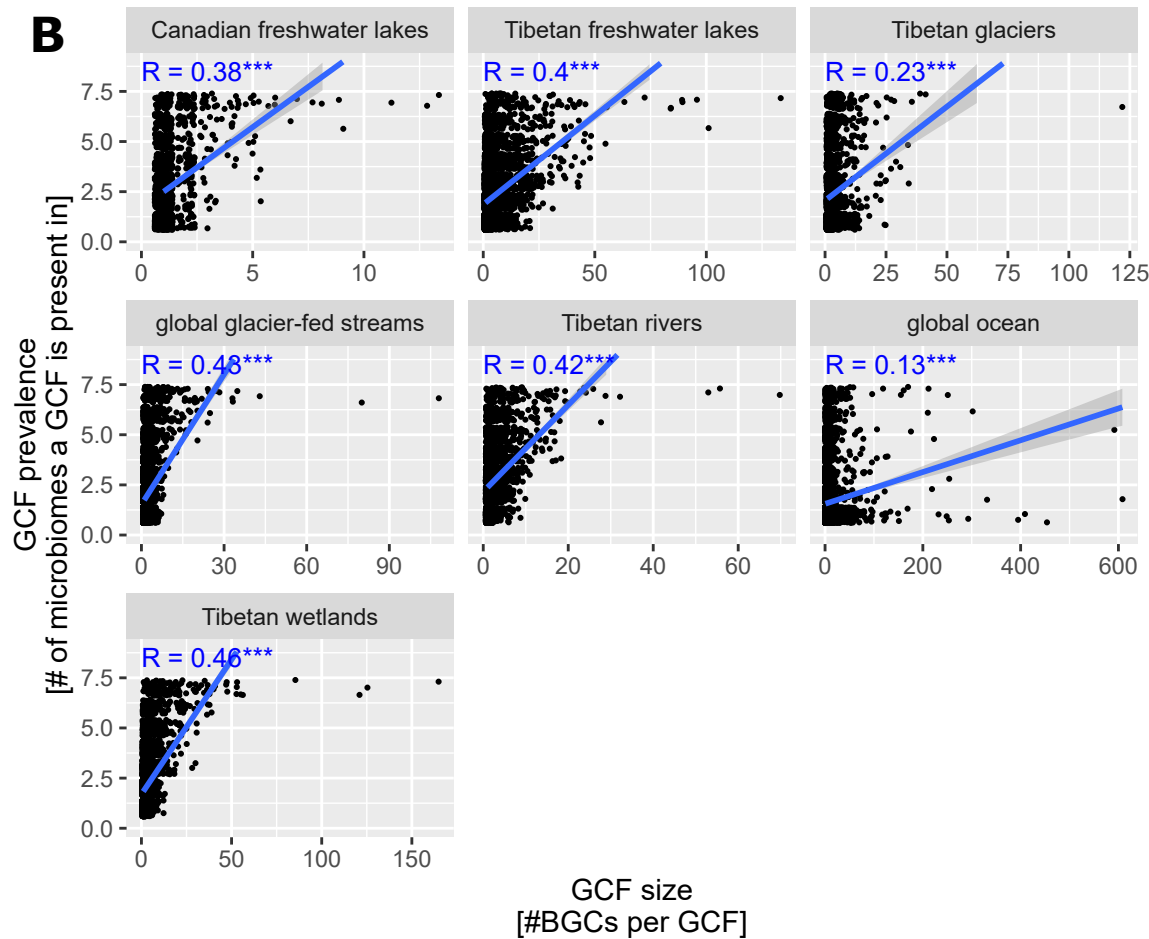

Supplement: Figure S1 [file msystems.01137-24-s0007.pdf]

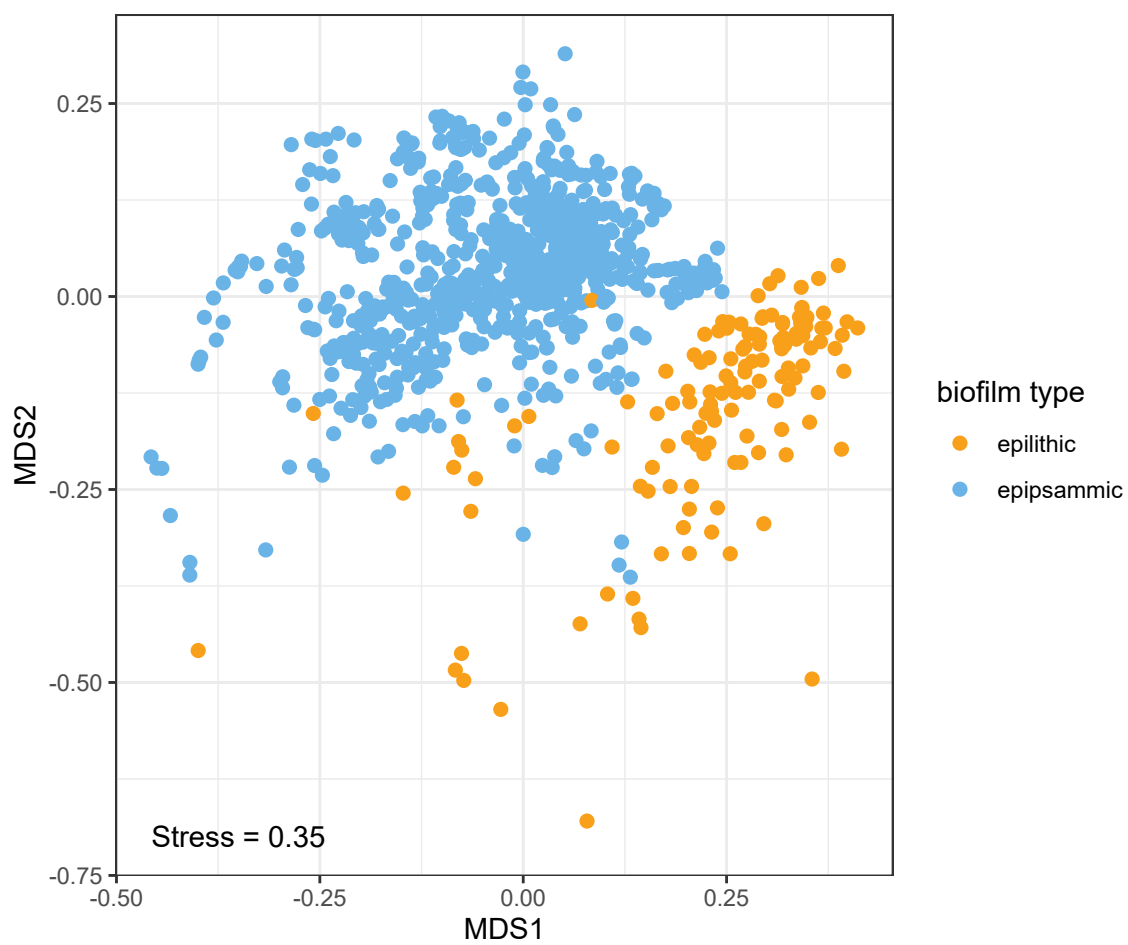

Supplement: Figure S2 — NMDS of prokaryotes. [file msystems.01137-24-s0008.pdf]
