# Supplementary material for: Deciphering the biosynthetic landscape of biofilms in glacier-fed streams
Source: mSystems. 2024 Dec 31;10(2):e01137-24. doi: 10.1128/msystems.01137-24 (PMC11834409; doi:10.1128/msystems.01137-24)
Supplement: Figure S3 — Extended distance decay curves. [file msystems.01137-24-s0003.pdf]

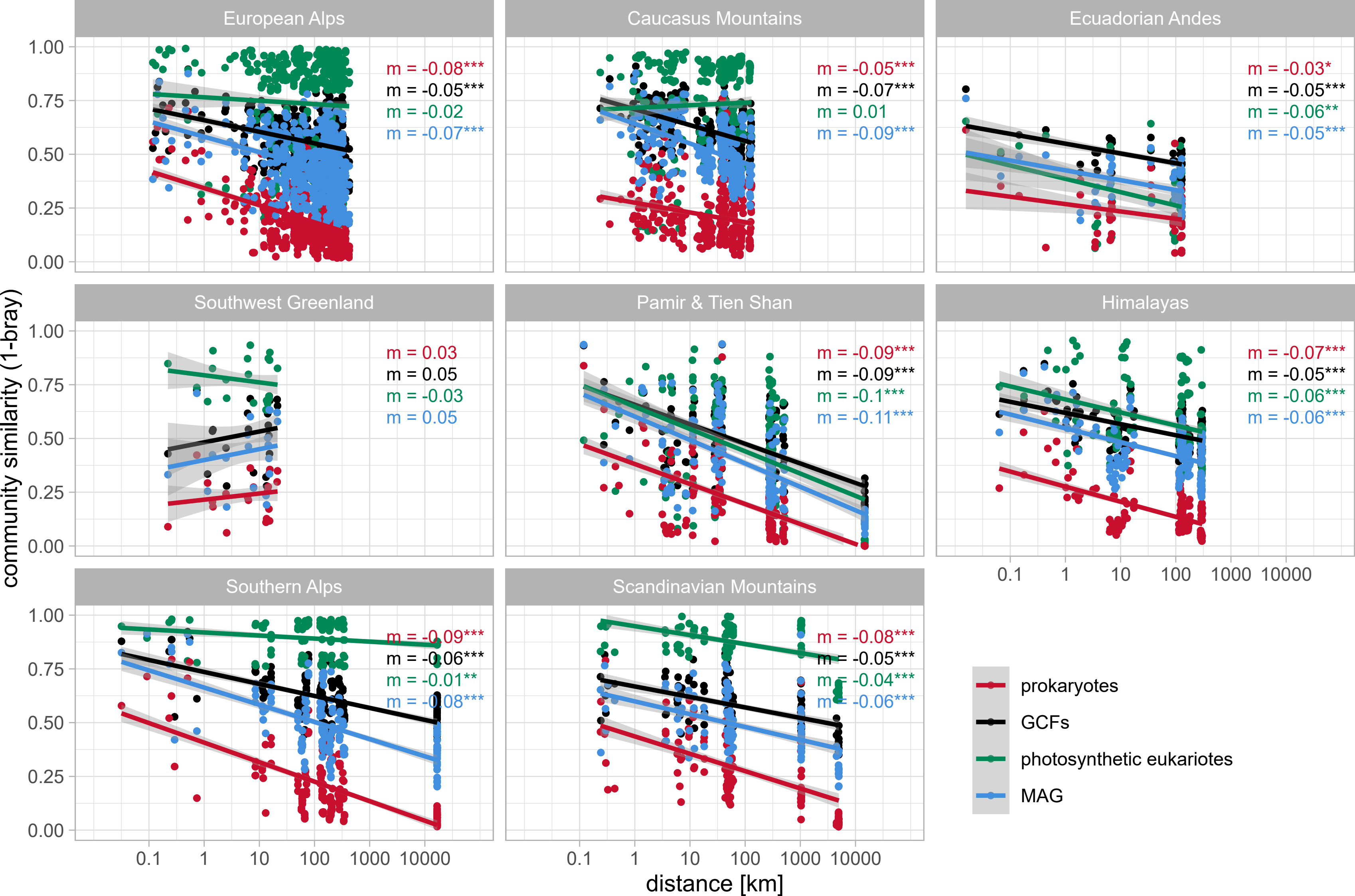
European Alps
m = -0.08***
m = -0.05***
m = -0.02
m = -0.07***
Caucasus Mountains
m = -0.05***
m = -0.07***
m = 0.01
m = -0.09***
Ecuadorian Andes
m = -0.03*
m = -0.05***
m = -0.06**
m = -0.05***
Southwest Greenland
m = 0.03
m = 0.05
m = -0.03
m = 0.05
Pamir & Tien Shan
m = -0.09***
m = -0.09***
m = -0.1***
m = -0.11***
Himalayas
m = -0.07***
m = -0.05***
m = -0.06***
m = -0.06***
Southern Alps
m = -0.09***
m = -0.06***
m = -0.01**
m = -0.08***
Scandinavian Mountains
m = -0.08***
m = -0.05***
m = -0.04***
m = -0.06***
community similarity (1-bray)
1.00
0.75
0.50
0.25
0.00
0.1
1
10
100
1000
10000
distance [km]
prokaryotes
GCFs
photosynthetic eukariotes
MAG
