# Supplementary material for: Deciphering the biosynthetic landscape of biofilms in glacier-fed streams
Source: mSystems. 2024 Dec 31;10(2):e01137-24. doi: 10.1128/msystems.01137-24 (PMC11834409; doi:10.1128/msystems.01137-24)
Supplement: Supplemental material — Tables S2 to S5 and legends for other supplemental files. [file msystems.01137-24-s0004.docx]

**Supplemental material**

**Supplementary table 1.**

List of all BGCs found in glacier-fed streams. Listed are also in which MAG and its taxonomy the BGCs was detected, the Gene cluster family the BGC is part of, the BGC category, the predicted product by BiG-SCAPE, the Euclidean distance to known BGCs in NCBI determined using BiG-SliCE, if the BGC lies on a contig edge and the length of the BGC.

**Supplementary table 2.** Percentages of the detailed BGC categories for the individual phyla.

| BGC category | Myxococcota | Elusimicrobiota | Fibrobacterota | Eremiobacterota | Planctomycetota | Verrucomicrobiota | Cyanobacteria | Deinococcota | Acidobacteriota | Desulfobacterota | Nitrospirota | Proteobacteria | Gemmatimonadota | Bdellovibrionota | Actinobacteriota | Bacteroidota | Chloroflexota | Armatimonadota | Eisenbacteria | Patescibacteria |
| --- | --- | --- | --- | --- | --- | --- | --- | --- | --- | --- | --- | --- | --- | --- | --- | --- | --- | --- | --- | --- |
| Terpene | 15.4 | 37 | 29.7 | 54.5 | 41 | 40.4 | 24.4 | 25 | 28.7 | 12.8 | 28.6 | 27.6 | 34.4 | 17 | 31.6 | 36.3 | 30.3 | 34.3 | 43.5 | 35.1 |
| RiPPs | 35.3 | 29.6 | 29.7 | 18.2 | 10.3 | 12.5 | 17.5 | 45 | 26.9 | 10.6 | 32.7 | 29 | 24.2 | 18 | 26.2 | 12.6 | 15.8 | 24.3 | 8.7 | 35.1 |
| PKS | 12.3 | 18.5 | 21.6 | 9.1 | 27.7 | 13.8 | 6 | 20 | 9.3 | 14.9 | 2 | 8.1 | 8.6 | 27.5 | 15.1 | 24.5 | 30.3 | 7.1 | 39.1 | 0 |
| NRPS | 18.1 | 7.4 | 0 | 9.1 | 12.6 | 14.8 | 38.7 | 5 | 28.5 | 8.5 | 10.2 | 10.2 | 21 | 6.9 | 12.4 | 6.5 | 14.5 | 18.6 | 8.7 | 5.4 |
| arylpolyene | 6.4 | 7.4 | 8.1 | 0 | 3.5 | 6.9 | 0 | 0 | 1.1 | 25.5 | 6.1 | 9.5 | 0.5 | 6.9 | 0.2 | 9.9 | 0 | 0 | 0 | 0 |
| acyl_amino_acids | 1.3 | 0 | 0 | 0 | 0.3 | 0.2 | 0 | 1.7 | 0.4 | 0 | 0 | 3 | 0 | 13.7 | 0 | 0.4 | 0 | 0 | 0 | 18.9 |
| hserlactone | 0.2 | 0 | 2.7 | 0 | 0.2 | 0 | 0 | 0 | 0.2 | 2.1 | 10.2 | 4.2 | 0 | 0.3 | 0 | 0 | 0 | 0 | 0 | 2.7 |
| PKS-NRP_Hybrids | 1.6 | 0 | 0 | 9.1 | 1 | 3.1 | 6 | 0 | 1.1 | 0 | 2 | 2 | 8.6 | 2 | 1.6 | 1.1 | 3.9 | 7.1 | 0 | 0 |
| resorcinol | 0.4 | 0 | 2.7 | 0 | 0.8 | 0.2 | 3.7 | 0 | 0.7 | 8.5 | 0 | 0.6 | 0.5 | 2.6 | 0 | 2.7 | 1.3 | 0 | 0 | 0 |
| phosphonate | 2.1 | 0 | 0 | 0 | 0.7 | 0 | 0 | 0 | 0 | 2.1 | 8.2 | 0.2 | 0 | 0.3 | 0 | 0 | 0 | 0 | 0 | 0 |
| betalactone | 0.4 | 0 | 0 | 0 | 0.3 | 0.5 | 0.5 | 0 | 1.1 | 4.3 | 0 | 3.4 | 0 | 0.7 | 7.1 | 1.2 | 0 | 2.9 | 0 | 0 |
| ladderane | 2.5 | 0 | 5.4 | 0 | 0.2 | 5.7 | 0 | 0 | 1.1 | 4.3 | 0 | 0 | 0 | 0 | 0.4 | 0.1 | 0 | 2.9 | 0 | 0 |
| resorcinol.arylpolyene | 0.1 | 0 | 0 | 0 | 0.2 | 0.2 | 0 | 0 | 0 | 4.3 | 0 | 0.3 | 0.5 | 1 | 0 | 4 | 0 | 0 | 0 | 0 |
| ectoine | 0 | 0 | 0 | 0 | 0 | 0 | 0 | 1.7 | 0 | 0 | 0 | 0.1 | 0 | 0 | 2.7 | 0 | 1.3 | 0 | 0 | 0 |
| betalactone.arylpolyene | 0 | 0 | 0 | 0 | 0 | 0 | 0 | 0 | 0 | 2.1 | 0 | 0 | 0 | 0 | 0 | 0 | 0 | 0 | 0 | 0 |
| other | 0 | 0 | 0 | 0 | 0.2 | 0.5 | 0 | 0 | 0 | 0 | 0 | 0 | 0 | 0 | 2 | 0.2 | 1.3 | 0 | 0 | 0 |
| indole | 1.4 | 0 | 0 | 0 | 0 | 0 | 1.8 | 0 | 0 | 0 | 0 | 0.4 | 0 | 0 | 0 | 0.1 | 1.3 | 0 | 0 | 0 |
| ectoine.terpene | 0 | 0 | 0 | 0 | 0 | 0 | 0 | 1.7 | 0 | 0 | 0 | 0 | 0 | 0 | 0 | 0 | 0 | 0 | 0 | 0 |
| siderophore | 0.4 | 0 | 0 | 0 | 0 | 0 | 0 | 0 | 0 | 0 | 0 | 0.2 | 1.1 | 1.6 | 0 | 0.2 | 0 | 0 | 0 | 1.4 |
| nucleoside | 0 | 0 | 0 | 0 | 0 | 0 | 0 | 0 | 0 | 0 | 0 | 0 | 0 | 0 | 0 | 0 | 0 | 1.4 | 0 | 0 |
| oligosaccharide | 0 | 0 | 0 | 0 | 0.2 | 0.2 | 0 | 0 | 0 | 0 | 0 | 0 | 0 | 0 | 0 | 0 | 0 | 1.4 | 0 | 0 |
| terpene.NRPS-like | 0.2 | 0 | 0 | 0 | 0 | 0 | 0 | 0 | 0.4 | 0 | 0 | 0 | 0 | 0 | 0 | 0 | 0 | 0 | 0 | 1.4 |

**Supplementary table 3.**

Summary of MAGs and BGCs used in this study and their respective references.

| **Dataset** | **reference** | **# MAGs** | **# BGCs** |
| --- | --- | --- | --- |
| Glacier-fed streams | Michoud et al. (7) | 2868 | 8040 |
| Tibetan glaciers | Liu et al. (39) | 1797 | 7066 |
| Canadian freshwater lakes | Garner et al. (43) | 1008 | 1843 |
| Global ocean | Paoli et al. (32) | 26,293 | 38,232 |
| Tibetan wetlands, rivers & lakes | Cheng et al. (42) | 10,723 | 44,349 |

**Supplementary table 4.**

Number of samples for the different datasets used in this study. Samples with less than 20’000 reads for 16S amplicons were removed and samples with less than 10’000 reads for 18S amplicons were removed, independently. The MAGs were also used in a study by Michoud et al. (7) and the 16S amplicons were used in a study by Ezzat et al. (65).

| **Dataset** | **# Streams** | **# Samples** | **# Samples including replicates** |
| --- | --- | --- | --- |
| GCF and MAG abundances Epipsammic | 85 | 153 | 156 |
| GCF and MAG abundances Epilithic | 5 | 17 | 17 |
| 16S amplicons Epipsammic | 157 | 298 | 830 |
| 16S amplicons Epilithic | 44 | 59 | 135 |
| 18S amplicons Epipsammic | 137 | 259 | 735 |
| 18S amplicons epilithic | 60 | 86 | 233 |

**Supplementary table 5.**

The normalized and summed abundance of all GCF belonging to six investigated secondary metabolites. Displayed is the averaged abundance across all epipsammic or epilithic samples respectively, and if they significantly vary (t-test adjusted p-value).

| **Secondary metabolite GCF** | **Epipsammic abundance (mean ± SD)** | **Epilithic abundance (mean ± SD)** | **p. adj** |
| --- | --- | --- | --- |
| Hserlactone | 243.6 ± 70.7 | 116.6 ± 108.2 | 0.004 |
| Cyclic lactone autoinducer | 7.0 ± 5.2 | 0.8 ± 1.4 | 1.2 x 10 ^-11^ |
| Arylpolyene | 586.4 ± 179.4 | 250.0 ± 154.1 | 3.70 x 10^-05^ |
| Ectoine | 15.7 ± 8.6 | 7.2 ± 10.5 | 0.029 |
| Siderophores | 6.9 ± 7.8 | 0.1 ± 0.3 | 4.6 x 10^-19^ |
| Vibrioferrins | 7.2 ± 5.2 | 8.3 ± 16.7 | 0.83 |

**Supplementary table 6.**

List of all sampled glacier-fed streams in the Vanishing Glaciers project, their coordinates and elevation at the snout and sample sites.

**Supplementary Figure 1. (A)** Jaccard distances of the investigated microbiomes based on the presence and absence of GCFs. **(B)** Associations between the GCF size (i.e. the number of BGCs that are within this GCF) and its prevalence (i.e. the number of microbiomes the GCF can be found in) for each of the investigated microbiome.

**Supplementary Figure 2.** NMDS plot of prokaryotic abundances based on 16S rRNA ASVs using Bray-Curtis distances, coloured by biofilm type.

**Supplementary Figure 3.** Distance-decay curves of biosynthetic, photosynthetic eukaryotes and prokaryotic (both based on 16S ASVs and MAGs) diversity. The respective community similarity (1 - Bray Curtis dissimilarity) is plotted against the distances between samples. (Epipsammic samples only). The slope (m) of linear model between the community similarity and the distance is displayed in the top right corner, with the adjusted p-value of the correlation denoted by stars (*** p < 0.001, ** p = 0.001 - 0.01, * p = 0.01 - 0.05).
